# Identification of Novel Genomic Islands in Liverpool Epidemic Strain of *Pseudomonas aeruginosa* Using Segmentation and Clustering

**DOI:** 10.3389/fmicb.2016.01210

**Published:** 2016-08-03

**Authors:** Mehul Jani, Kalai Mathee, Rajeev K. Azad

**Affiliations:** ^1^Department of Biological Sciences, University of North TexasDenton, TX, USA; ^2^Department of Human and Molecular Genetics, Herbert Wertheim College of Medicine Global Health Consortium, and Biomolecular Sciences Institute, Florida International UniversityMiami, FL, USA; ^3^Department of Mathematics, University of North TexasDenton, TX, USA

**Keywords:** genomic islands, pathogen evolution, *Pseudomonas aeruginosa*, Liverpool epidemic strain, cystic fibrosis, virulence, antibiotic resistance, genome segmentation

## Abstract

*Pseudomonas aeruginosa* is an opportunistic pathogen implicated in a myriad of infections and a leading pathogen responsible for mortality in patients with cystic fibrosis (CF). Horizontal transfers of genes among the microorganisms living within CF patients have led to highly virulent and multi-drug resistant strains such as the Liverpool epidemic strain of *P. aeruginosa*, namely the LESB58 strain that has the propensity to acquire virulence and antibiotic resistance genes. Often these genes are acquired in large clusters, referred to as “genomic islands (GIs).” To decipher GIs and understand their contributions to the evolution of virulence and antibiotic resistance in *P. aeruginosa* LESB58, we utilized a recursive segmentation and clustering procedure, presented here as a genome-mining tool, “GEMINI.” GEMINI was validated on experimentally verified islands in the LESB58 strain before examining its potential to decipher novel islands. Of the 6062 genes in *P. aeruginosa* LESB58, 596 genes were identified to be resident on 20 GIs of which 12 have not been previously reported. Comparative genomics provided evidence in support of our novel predictions. Furthermore, GEMINI unraveled the mosaic structure of islands that are composed of segments of likely different evolutionary origins, and demonstrated its ability to identify potential strain biomarkers. These newly found islands likely have contributed to the hyper-virulence and multidrug resistance of the Liverpool epidemic strain of *P. aeruginosa*.

## Introduction

*Pseudomonas aeruginosa* dwells in diverse environments including soil, water, and air. *P. aeruginosa* strains have also been isolated from medical equipment such as catheters (Nickel et al., 1985). *P. aeruginosa* strains have multifarious metabolic capabilities, including the ability to degrade gasoline, kerosene, and diesel (Wongsa et al., 2004). As a pathogen, *P. aeruginosa* infects a broad range of host organisms, from vertebrates to non-vertebrates (Rahme et al., 2000). In humans, *P. aeruginosa* causes post-operative infections as well as infections in other immuno-compromised conditions, such as burn wounds and cancer (Sato et al., 1988; Rolston and Bodey, 1992). It is well known for causing morbidity and mortality in patients with cystic fibrosis (CF; Emerson et al., 2002). In most CF cases, *P. aeruginosa* is known to cause persistent respiratory infections that lead to untimely mortality (Cystic Fibrosis Foundation Report).

Comparative genome studies have revealed a high level of conservation among *P. aeruginosa* strains, with sequence diversity limited to about 10% of the pan-genome (Spencer et al., 2003; Lee et al., 2006; Cramer et al., 2011). These regions, collectively referred to as the “accessory” genome (Mathee et al., 2008), have an abundance of antibiotic resistance, virulence and biofilm-associated genes. Often these genes are acquired as clusters of functionally related genes from distantly related or unrelated organisms through the evolutionary process of horizontal gene transfer (HGT; Mathee et al., 2008; Klockgether et al., 2011). These HGT-acquired gene clusters, referred to as “genomic islands (GIs),” are interspersed in the regions of genome plasticity flanked by highly conserved sections of *P. aeruginosa* genomes (Lee et al., 2006; Mathee et al., 2008; Winstanley et al., 2009; Roy et al., 2010).

An important goal in pathogen genomics is to identify and characterize GIs in pathogens and assess their virulence and antibiotic resistance potential. Both bottom-up and top-down methods are employed to catalog GIs in the bacterial genomes. Bottom-up methods, either gene based or moving-window based, are designed to first identify the atypical genes or windows which are then grouped into GIs based on their physical association (Arvey et al., 2009). However, because of the variable compositional character of GIs, weakly atypical genes or windows are often misclassified, resulting in predictions that are actually the fragments of GIs. This also complicates the delineation of island boundaries. The gene-based database-dependent methods, such as *SIGI* (Waack et al., 2006), are limited in their ability to classify orphan genes. The window-based methods, such as IVOM (Vernikos and Parkhill, 2006), are designed to be independent of databases, however, they have inherited the weaknesses of the moving-window approach– smaller window size increases stochastic fluctuations while larger size diminishes resolution. Moreover, the bottom-up methods are inherently limited in their ability to precisely delineate the alien and native regions.

In contrast, the top-down methods start with the entire genome and progressively divide it into smaller segments to localize regions of atypical composition (Arvey et al., 2009). A frequently invoked top-down procedure entails fragmenting a genomic sequence recursively using the Jensen–Shannon divergence measure generalized within a Markov chain model framework (Markovian Jensen–Shannon Divergence or MJSD) until the segments are rendered compositionally homogeneous within but heterogeneous between (Azad and Li, 2013). This is followed by an agglomerative, non-hierarchical clustering process to group compositionally similar segments (Azad and Li, 2013). This procedure segregates genomic segments from different sources efficiently, thus revealing the mosaic compositional structure of the genomes. This method was earlier assessed on an artificial chimeric genome of *Escherichia coli* with ~25% genes acquired from 10 donors as well as on the well-studied, genuine bacterial genomes (Azad and Li, 2013). The MJSD-based segmentation–clustering method (Azad and Li, 2013) outperformed other existing methods (Azad and Li, 2013), including RHOM that is based on a hidden Markov model (Nicolas et al., 2002), a Bayesian method (Boys and Henderson, 2004), and an optimization method (K-H segmentation; Gionis and Mannila, 2003).

The MJSD-based segmentation–clustering method, by virtue of its ability to classify genes by their ancestry, can decipher the mosaic structure of GIs. GIs are called mosaic if they are composed of segments of different ancestries. Thus, in addition to deciphering the mosaic compositional structure of a genome (Azad and Li, 2013), this method can be used to examine the fine-scale structures, such as the mosaic structure of GIs arising because of the serial acquisition of DNAs of different ancestries at a genomic locus. The MJSD-based method can localize even genomic islets (Hacker and Kaper, 2000), which are relatively small segments (size < 10 Kbp) acquired through HGT. In this paper, we use a modified version of this method (Azad and Li, 2013), “GEMINI,” named after its genome mining function, to identify novel GIs in *P. aeruginosa*. In this study, the GEMINI program was applied to a transmissible *P. aeruginosa* strain (Cheng et al., 1996).

Prior to 1996, it was believed that CF patients acquire only non-transmissible, unique strains of *P. aeruginosa* from the environment (Cheng et al., 1996). This idea was debunked with the isolation of an epidemic strain from CF children in Liverpool, UK, and hence, is referred to as the “Liverpool epidemic strain” or LES (Cheng et al., 1996). In the last decade, many new epidemic strains of *P. aeruginosa* have been discovered across the globe (Jones et al., 2001; Armstrong et al., 2003; O’Carroll et al., 2004; Scott and Pitt, 2004; Lewis et al., 2005; Bradbury et al., 2008; Aaron et al., 2010). Apart from being transmissible, the LES strain causes high morbidity and greater loss of lung function in CF patients as compared to non-LES strains (Al-Aloul et al., 2004). The LES strain has also been implicated in renal failure in adult CF patients (Al-Aloul et al., 2005). In addition, this strain can cause cross-infections; in one such case, healthy parents were infected by LES from a CF patient (McCallum et al., 2002). Many variants of LES epidemic strain of *P. aeruginosa* have since been identified (Jones et al., 2001; Armstrong et al., 2003; Bradbury et al., 2008; Aaron et al., 2010). One of these, LESB58 strain, possesses almost all known virulence genes of *P. aeruginosa* (Winstanley et al., 2009). Although the original LESB58 isolates were sensitive to some antibiotics, it has become difficult to eradicate LESB58 from the lungs of CF patients once the infection has been established (Winstanley et al., 2009). Except for PA2818, a putative aminoglycoside response regulator (*arr*), all other antibiotic resistance and susceptibility genes found in *P. aeruginosa* PAO1 are present on the chromosome of LESB58 (Winstanley et al., 2009).

The propensity of the *P. aeruginosa* LES strain, especially LESB58, in colonizing the lungs of CF patients could likely be due to the accessory genes resident on GIs. Though several LESB58 GIs have been reported (Winstanley et al., 2009), a comprehensive analysis of the LESB58 genome that could shed light on its epidemic traits has not yet been performed. In pursuance of our goal to leverage the augmented power of a proven integrated segmentation and clustering approach (Azad and Li, 2013) to probe the genome of a pathogen for yet uncharacterized genomic or pathogenicity islands, we revisited the genome of *P. aeruginosa* LESB58 with GEMINI to decode its virulence potential and understand its unique traits in light of its evolution via HGT. We also deconstructed the mosaic GIs to understand their contributions to the LESB58 pathogenicity. The predicted islands were further compared with other sequenced *P. aeruginosa* genomes using sequence alignment approaches. This study unraveled the yet unknown genetic aspects underlying LESB58’s virulence and resistance traits.

## Materials and Methods

### Genome Sequences

The genome sequences of 11 representative *P. aeruginosa* strains, namely, LESB58 (NCBI Accession: NC_011770.1; Winstanley et al., 2009), PACS2 (Accession: NZ_AAQW01000001.1; Wiehlmann et al., 2007), SCV20265 (Accession: NC_023 149.1; Eckweiler et al., 2014), PA2192 (Accession: NZ_CH482384.1; Mathee et al., 2008), PA7 (Accession: NC_0096 56.1; Roy et al., 2010), PA14 (Accession: NC_008463.1; Lee et al., 2006), C3719 (Accession: NZ_CH482383.1; Mathee et al., 2008), PA1 (Accession: NC_022808.2; Lu et al., 2015), B136-33 (Accession: NC_020912.1^1^), PAO1 (Accession: NC_002516.2; Winsor et al., 2011), C7447M (Accession: NC_022360.1; Yin et al., 2013), and the gene coordinates were obtained from the NCBI ftp site and *Pseudomonas* database (Winsor et al., 2009). Of these, LESB58 and C3719 are the epidemic strains (Cheng et al., 1996; Jones et al., 2001).

### Genomic Island Detection Using GEMINI

The genome mining tool is a next generation tool that utilizes segment context information within an integrated segmentation and clustering framework to robustly identify GIs. The integrated segmentation and clustering method was proposed earlier for genomic data interpretation, including alien segment localization (Azad and Li, 2013). Briefly, within this framework, a genome sequence is subjected to a recursive binary segmentation. The segmentation procedure splits the genome into two parts at a position where the compositional difference between the resulting sequence segments is maximized [assessed using an information-entropy based divergence measure, generalized with the framework of Markov model (Thakur et al., 2007; Arvey et al., 2009)], provided this difference be large enough to be statistically significant (assessed using the probability distribution of maximum value of the divergence measure in random sequences). This is followed recursively for each resulting sequence segment. The recursive process is halted when each segment becomes compositionally homogeneous to be fragmented further at a pre-specified level of statistical significance. An agglomerative clustering procedure follows in two steps. First, the contiguous compositionally similar segments, which may arise because of hyper-segmentation, are identified and grouped together. This is followed by recursively grouping the similar clusters within the same framework of statistical hypothesis testing, which entails testing the null hypothesis that the two sequence segments are compositionally similar (Azad and Li, 2013; Jani and Azad, 2013).

The above procedure creates several clusters, with the largest harboring between ~50–95% of the genes [native genes being the most abundant that is consistent with previous studies (Lawrence and Ochman, 1998; Garcia-Vallve et al., 2003)] and the other smaller clusters harboring genes that are likely of foreign origin. Each segment is labeled as native or alien depending on the cluster it belongs to. The contiguous alien segments are assembled as putative GIs. We modified the clustering procedure to augment the power of the MJSD-based method in identifying the GIs. This modification entails retrieving clusters at a strict clustering stringency that minimizes merger of segments or clusters from disparate sources. This, however, results in multiple clusters for native segments. The strongly typical native segments form a large cluster (typically, >50% of a genome) whereas weakly typical native segments group into one or more smaller clusters. Allowing merger of these smaller clusters into the largest cluster by relaxing the stringency may result in potentially unwanted merger of alien clusters into the native clusters. We therefore invoked the segment context information to minimize the false positive and false negative error rates by allowing merger of truly native clusters while precluding undesirable mergers. We took two cues from the segment context information to merge native clusters. Because the native segments falling into different clusters owing to their compositional variability are expected to be physically associated at genomic loci, their contexts (neighboring segments) will more likely be lying within the native clusters. And, second, the stochastic variations resulting in compositionally ambiguous native segments should also result in sporadic distribution of such segments within the genome. Therefore, at a conservative clustering setting, the compositionally ambiguous native segments should appear more sparsely distributed or scattered in comparison to the rather clustered landscape of other segment types within the genome. GEMINI uses these cues to identify clusters representing the native genome.

### Other GI Detection Methods

Genome mining tool was assessed against several current methods for identification of a set of verified GIs from two strains of *P. aeruginosa*, namely, LESB18 and PA14. These methods are described briefly below.

#### IslandPath

This visualization tool presents a gene map where the potential GI harbored genes, inferred by their significant difference from the G+C content and dinucleotide composition of the genome, and GI specific features such are tRNA genes are indicated by special colors markings (Hsiao et al., 2003).

#### IslandPath-DIMOB

IslandPath-DIMOB identifies GIs through their dinucleotide bias and the presence of mobility genes (Hsiao et al., 2003).

#### Score Based Identification of Genomic Island Using Hidden Markov Models (SIGI-HMM)

Score based identification of genomic island using hidden Markov models (SIGI-HMM) uses the codon usage frequency table of organisms as their distinctive signatures (Waack et al., 2006). The SIGI-HMM procedure exploits the difference in codon usage bias between recipient and donor organisms to infer putatively alien DNAs.

#### IslandPick

This is an automated comparative genomics method that selects related genomes for a given query (Langille et al., 2008) and then perform whole genome alignment using MAUVE (Darling et al., 2004).

#### IslandViewer

Outcomes of three GI prediction programs, IslandPath-DIMOB, SIGI-HMM, and IslandPick are integrated by IslandViewer into a web interface (Langille and Brinkman, 2009). It reports better accuracy than any of these three methods.

#### Interpolated Variable Order Motif (IVOM; Alien Hunter)

Interpolated Variable Order Motif (IVOM) measures the difference in compositional bias between a sequence in a moving window and the genome within the framework of an interpolated Markov model framework (Vernikos and Parkhill, 2006). The contiguous atypical regions are annotated GIs.

#### Markovian Jensen–Shannon Divergence (MJSD)

This method uses Markov model to assess the atypicality of genomic segments, obtained via recursive segmentation, against the genome background (Arvey et al., 2009).

#### Zisland Explorer

This new tool scans a genome for “leaps” in cumulative GC profile; these atypical regions are annotated GIs (Wei et al., 2016).

These parametric methods, with the exception of MJSD and Zisland Explorer, invoke bottom-up approaches, initially classifying genes or sequence windows as alien or native, and then assembling them into GIs or the native genome.

### Phylogenetic Analysis

Phyletic pattern of the predicted island-borne genes were analyzed by examining their presence or absence in the genomes of close relatives of *P. aeruginosa* LESB58. Atypical distribution of orthologous genes, i.e., the absence of a gene of interest from the genomes of closely relatives, provides an evidence in support of acquisition of the gene by HGT. The sequence comparison was performed via BLAST (Altschul et al., 1990) to screen unusual phyletic pattern in the distribution of the predicted island-borne genes. Initially, the BLAST search was restricted to the *P. aeruginosa* group (NCBI taxid: 136841) to identify genes present among the strain relatives of LESB58. This was followed by restricting the search to *Pseudomonadaceae* family (NCBI taxid: 135621) excluding the *P. aeruginosa* group. This helped in assessing the distribution of alien genes in all the members of the family the LESB58 belongs to. The distribution of a putative alien gene was considered atypical if the majority of the LESB58’s close relatives do not carry it.

## Results

In this study, we applied GEMINI to deconstruct the genome of an epidemic strain *P. aeruginosa* LES58 (Cheng et al., 1996). The reasons for employing GEMINI as the method and *P. aeruginosa* LESB58 as the model organism were manifold: (a) There have been reports on hyper-virulence arising because of the presence of hybrid pathogenicity islands in the bacterial pathogens (He et al., 2004). Although the original MJSD-based segmentation and clustering method was shown to be effective in localizing the GIs (Azad and Li, 2013), its ability to decipher the mosaic structure of GIs was never harnessed for understanding the contribution of the mosaicism in pathogenicity. The modified program GEMINI was therefore applied to the genome of a hyper-virulent strain *P. aeruginosa* LESB58 (Cheng et al., 1996). We hypothesized that the unique mosaic GIs in this strain would be unraveled using GEMINI. (b) Previous studies had validated four GIs in LESB58 using wet-lab assays, and therefore, this genome provided an opportunity to benchmark GEMINI against the other GI prediction methods. (c) The availability of a large number of closed genomes provided an opportunity to compare with the first identified epidemic *P. aeruginosa* isolate, the LESB58 strain, in order to assess the genotypic differences between the epidemic and non-epidemic strains.

### Comparative Assessment of GI Prediction Methods

We used second order MJSD (*m* = 2 in Eq. 1) in GEMINI that generated 446 segments when applied to the 6.6 Mbp *P. aeruginosa* LES58 genome. These segments were grouped into 25 clusters with the largest cluster harboring 246 strongly typical native segments, while 102 weakly typical native segments grouped into a separate cluster. These two clusters were merged into a single native cluster using segment context information. The predicted alien segments were assigned to 23 clusters with the largest alien cluster harboring 61 segments and the rest containing much fewer (<10) segments. Segments resident in the alien clusters were labeled GIs if they harbored eight or more contiguous genes; otherwise they were called genomic islets. Contiguous atypical segments belonging to different alien clusters were collectively annotated as a mosaic GI. The compositionally distinct segments within a mosaic GI likely represent different ancestries. Although the parametric methods have been useful in predicting alien DNAs, occasionally native genes of unusual composition, such as those with high expression, may be misclassified as alien, resulting in false positives. These misclassified native genes have atypical codon usage not representative of the bulk of the native or ancestral genes (Sharp and Li, 1987; Langille et al., 2010). We, therefore, examined the predicted islands for the presence of highly expressed genes including those that encode ribosomal proteins. The predicted islands with abundance of these genes were reassigned to the core genome (Karlin, 2001). The performance of GEMINI in identifying GIs was assessed on an island-rich region in the *P. aeruginosa* LESB58 genome (~2.5–2.9 Mb; Langille et al., 2010), and was compared with that of MJSD (Arvey et al., 2009), IVOM (Vernikos and Parkhill, 2006), IslandPath-DIMOB (Hsiao et al., 2005), Island Viewer (Langille and Brinkman, 2009), Zisland Explorer (Wei et al., 2016), and SIGI HMM (Waack et al., 2006). Some of these methods had earlier been used to annotate GIs in the *P. aeruginosa* LESB58 genome (Langille et al., 2010).

**FIGURE 1:**
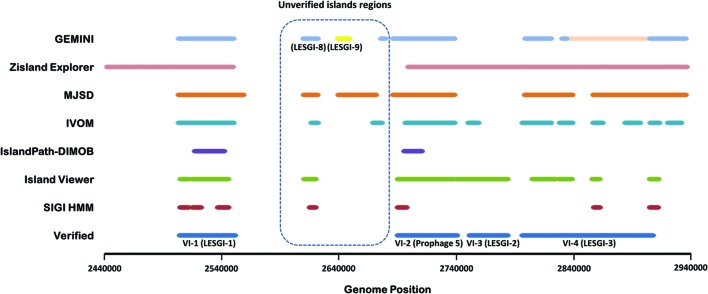
**Assessment of the methods for genomic island prediction on validated islands localized in an island-rich region in the *P. aeruginosa* LESB58 genome.** The performances of commonly used GI prediction tools, MJSD (Arvey et al., 2009), IVOM (Vernikos and Parkhill, 2006), IslandPath-DIMOB (Hsiao et al., 2005), IslandViewer (Langille and Brinkman, 2009), and SIGI HMM (Waack et al., 2006) were compared with that of GEMINI in identifying four experimentally verified islands, VI-1 (2504700-2551100 bp), VI-2 (2690450-2740350 bp), VI-3 (2751800-2783500 bp), and VI-4 (2796836-2907406 bp; Langille et al., 2010). Unverified island region shows islands predicted computationally but not verified experimentally.

The island-rich region (~2.5–2.9 Mb) used for assessment contains four GIs that were confirmed by laboratory experiments (‘Verified Islands,’ VI-1 to VI-4; **Figure 1**) (Langille et al., 2010). The GI prediction by GEMINI and other methods in this region is shown in **Figure 1** that summarizes the strengths and weaknesses of different methods. Whereas some methods including SIGI HMM and IslandPath-DIMOB had very limited success in identifying these islands, missing either entire or parts of the islands, predictions by IVOM was highly fragmented similar to a previous study (Arvey et al., 2009). Several methods including GEMINI and IVOM localized VI-1 efficiently, however, their performance varied significantly in localizing the other three islands. While GEMINI and IVOM delineated VI-2 efficiently, both missed VI-3 entirely. In contrast, IslandViewer identified VI-3, however, both VI-2 and VI-3 were predicted as one island. The most recent published program Zisland Explorer could also identify VI-3, however, as a part of a prediction that also spanned VI-2 and VI-4. The largest verified island, VI-4, posed a significant challenge to all methods. As expected, being a large island harboring over 100 genes, VI-4 was picked more efficiently by segmentation methods including MJSD and GEMINI. Overall, in comparison to other methods, GEMINI was able to resolve with greater precision this large island. GEMINI, Zisland Explorer, MJSD, and IVOM concurred on extending the right boundary of VI-4; the ORFs in this region include those coding for tRNA-Cys, tRNA-Leu, a transcriptional regulator, a two-component system, and permease among others (Supplementary Table S1).

Genome mining tool also predicted three novel islands in an unverified region (**Figure 1**). Each of these islands were also predicted, in entirety or in part, by one or more of the other methods, namely, MJSD, IVOM, or SIGI-HMM. Two of these predicted islands were named as LESGI-8 and LESGI-9, and the third was found to be a part of known “Prophage 5” (discussed in details below).

**FIGURE 2:**
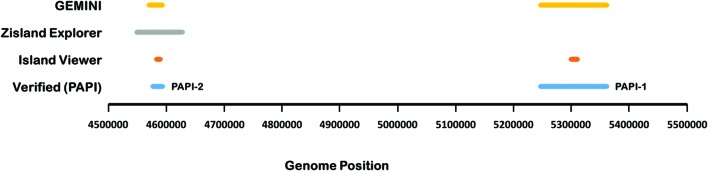
**Prediction of verified pathogenicity islands, PAPI-1 and PAPI-2, in *P. aeruginosa* PA14 by GEMINI, Zisland explorer (Wei et al., 2016), and Island Viewer (Dhillon et al., 2015).** GEMINI was implemented at the same parameter setting as for the LESB58 genome, and the native clusters were identified by comparing with the LESB58 clusters.

In addition, we assessed the performance of IslandViewer, Zisland Explorer, and GEMINI on verified islands in *P. aeruginosa* PA14, namely PAPI-1 and PAPI-2 (He et al., 2004; Qiu et al., 2006; Harrison et al., 2010) (**Figure 2**). Both PAPI-1 and PAPI-2 show characteristics of GI (He et al., 2004). PAPI-1 was shown to be transferrable between *P. aeruginosa* strains (Qiu et al., 2006). GEMINI compared favorably in identifying both of these islands.

### Novel Genomic Islands

**FIGURE 3:**
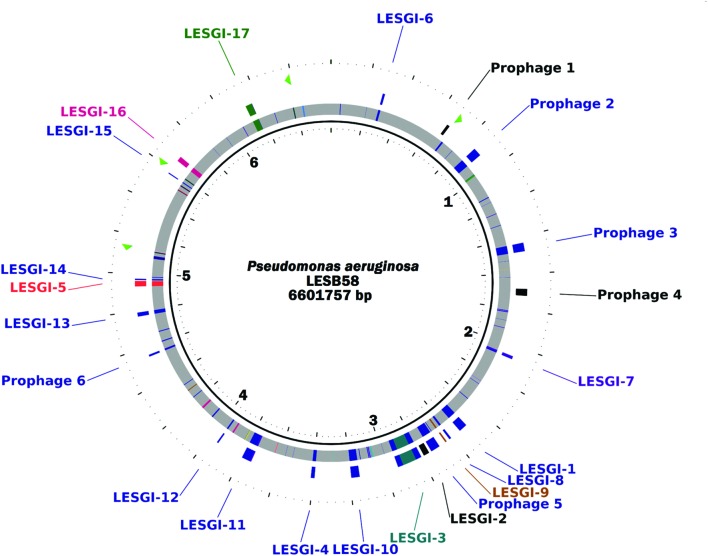
**Genomic islands in *P. aeruginosa* LESB58.** The innermost track shows the genomic segments in the native cluster in gray and those in the alien clusters in color. Different colors represent different alien clusters generated by the GEMINI program. The outer track shows the GEMINI predicted genomic islands as well as the previously reported islands, and the outermost track shows the location of rRNA operons. The genome map was generated using CGView (Stothard and Wishart, 2005). A second order Markov model was used by GEMINI for segmentation of the LESB58 genome and clustering of similar segments. The significance threshold for segmentation was set at 10^-10^. The clustering thresholds for two-step clustering were both set at 10^-13^.

Genome mining tool predicted 20 GIs (**Table 1**) of which eight overlapped with the previously reported islands, whereas 12 were novel. The segmental map of the LESB58 genome is shown in **Figure 3**, with alien segments (color-coded, each representing a distinct cluster) discriminated against the genome backbone (shown in gray). The GC content along the genome (Stothard and Wishart, 2005) is shown in Supplementary Figure S1, with known and novel GIs indicated for visualization against the GC landscape of the genome. The GC content and features of the novel GIs and that of the whole genome are given in Supplementary Table S2.

**Table 1:** Genomic islands in *P. aeruginosa* LESB58 genome.

Genomic island	Coordinate predicted by GEMINI		Length (bp)	Number of genes	Genes with aberrant phyletic pattern	Coordinates of previously reported islands (Winstanley et al., 2009)	
	Start	End				Start	End
LESGI-6	280017	292442	12425	9	0		
Prophage 1^∗^	–	–	14824			665561	680385
Prophage 2^∗∗^	860144	906115	45971	48	45	863875	906018
Prophage 3^∗∗^	1433751	1480246	46495	56	56	1433756	1476547
Prophage 4^∗^	–	–	36805			1684045	1720850
LESGI-7^∗∗^	2054683	2071280	16597	14	14		
LESGI-1	2503361	2549406	46400	32	32	2504700	2551100
LESGI-8	2609726	2621297	11571	13	0		
LESGI-9	2639569	2647891	8322	10	1		
Prophage 5^∗∗^	2686181	2737842	51661	69	61	2690450	2740350
LESGI-2^∗^	–	–	31700			2751800	2783500
LESGI-3^∗∗^	2798951	2935111	136160	125	100	2796836	2907406
LESGI-10^∗∗^	3147091	3192857	45766	13	13		
LESGI-4^∗∗^	3390565	3410660	20095	16	16	3392800	3432228
LESGI-11	3743159	3796926	53767	40	5		
LESGI-12	3945707	3954100	8393	8	0		
Prophage 6	4545171	4555810	10639	13	2	4545190	4552788
LESGI-13	4768774	4790400	21626	24	0		
LESGI-5^∗∗^	4931495	4960934	29439	25	25	4931528	4960941
LESGI-14	4965792	4973115	7323	9	0		
LESGI-15	5572987	5583916	10929	11	0		
LESGI-16	5656348	5683597	27249	28	0		
LESGI-17	6127068	6164062	36994	33	0		
Total			731151	596	370		

*GI coordinates are based on islands obtained after recursive segmentation and agglomerative clustering. Novel predictions have been highlighted. Phylogenetic support is based on BLAST analysis. Unusual phyletic pattern, indicating a recent island transfer into the LESB58 strain, was determined by examining the distributions of the LESB58 island borne genes in the genomes of P. aeruginosa strains. The distribution of a gene was considered unusual or atypical if the majority of the LESB58’s close relatives (other P. aeruginosa strains) do not carry it. ^∗^Previously reported LESB58 islands not predicted by GEMINI. ^∗∗^LESB58 islands showing unusual phyletic pattern*.

The novel islands were further analyzed to identify features commonly ascribed to GIs and for their distribution in other strains. Previous studies have reported 11 islands, annotated LESGI 1-5 and Prophage 1-6; we followed this nomenclature to name novel islands predicted by GEMINI. The criteria to characterize and discriminate GIs from other features in a genome are as follows (Langille et al., 2010; Che et al., 2014): (a) atypical composition, (b) presence of tRNA or tmRNA genes, which often serve as insertion sites, (c) presence of insertion sequence elements and direct repeats flanking GIs, (d) presence of mobility genes, such as, integrase and transposase genes, and (e) presence of virulence and antibiotic resistance genes (in pathogenic strains) or genes coding for novel metabolic traits. A GI may have one or more of these features. GIs may also have mosaic structure because of the acquisition of genomic segments from different sources at the same locus (Klockgether et al., 2004). In addition to examining these features, we also examined the phyletic pattern of GI-borne genes, which may have limited phylogenetic distribution (Langille et al., 2008).

Out of the total 596 genes identified on our predicted GIs, 370 genes show aberrant phyletic pattern, that is, they were absent in the genomes of a majority of the close relatives of *P. aeruginosa* LESB58. The phyletic distributions of these genes indicate recent HGT events that have shaped the genomes of *P. aeruginosa* strains. The set of the remaining 226 genes includes 22 tRNA genes which are known to be well-conserved across different strains and therefore do not show unusual phyletic pattern. Of the 20 GIs predicted by our method, nine islands showed unusual phyletic pattern, with majority of their genes having atypical distribution in the strains (**Table 1**). We describe below the GIs that were identified by GEMINI but were missed by other methods (**Table 1**).

### LESGI-6

**FIGURE 4:**
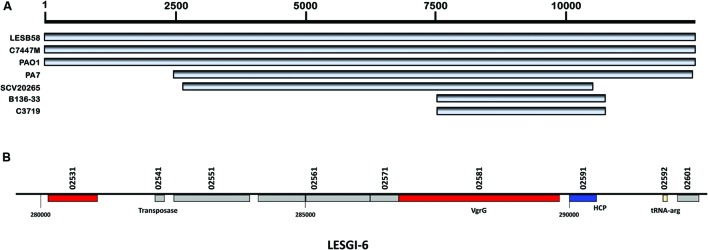
**Segmental and gene map of LESGI-6 (280017-292442 bp). (A)** LESGI-6 in *P. aeruginosa* LESB58 compared against the homologs in other *P. aeruginosa* strains using BLASTN. **(B)** Genes on LESGI-6: Signatures of transfer, namely, the phage and transposon genes – *vgrG*, *hcp*, *tnaA*, and tRNA are indicated.

Comparative analysis of the 11 *P. aeruginosa* strains (see Materials and Methods) revealed that this LESB58 island, LESGI-6, was found in its entirety in the C7447M and PAO1 strains, and in parts in PA7, SCV20265, B136-33, and C3719 strains (**Figure 4A**). LESGI-6 is either absent in the remaining strains or is just difficult to delineate in these strains because of extensive rearrangements (**Figure 4A**).

LESGI-6 harbors nine genes, *PALES_02531, PALES_02541, PALES_02551, PALES_02561, PALES_02571, PALES_02581, PALES_02591, PALES_02592*, and *PALES_02601* (Winsor et al., 2011) including a tRNA-Arg coding gene (**Figure 4B**). The protein-product of *PALES_02541* is 100% identical to the transposase of *P. aeruginosa* LES431 (NCBI protein id: YP_008940382.1). This island also harbors genes, namely *PALES_02581* and *PALES_02591*, encoding type VI secretion system (T6SS) core proteins. Haemolysin co-regulated protein (HCP) is encoded by *PALES_02591* and valine-glycine repeat protein G (VgrG) is encoded by *PALES_02581* (Winsor et al., 2011). Both HCP and VgrG show structural similarity to phage proteins; the HCP protein is related to tail protein of phage lambda, gpV (Mougous et al., 2006; Pell et al., 2009), and VgrG adopts a quaternary arrangement similar to gp27/gp5 complex of the bacteriophage T4 (Kanamaru et al., 2002; Leiman et al., 2009). Further, *PALES_02581* has four phage protein domains – a phage GPD domain, phage base V domain, T6SS Vgr domain and DUF2345. Importantly, the domain similar to the Vgr protein is integral to the function of T6SS. Interestingly, however, this island lacks the other T6SS genes. The roles of other genes on this island remain unclear.

The presence of homologous genes in other closely related strains makes it difficult to identify this island through comparative genomics approaches (**Table 1**). This island may have also been acquired by the common ancestor of *P. aeruginosa* or may have been transmitted to many *P. aeruginosa* strains since its acquisition. Notably, this region still bears atypical compositional characteristics, and could, therefore, be detected by GEMINI.

### LESGI-7

**FIGURE 5:**
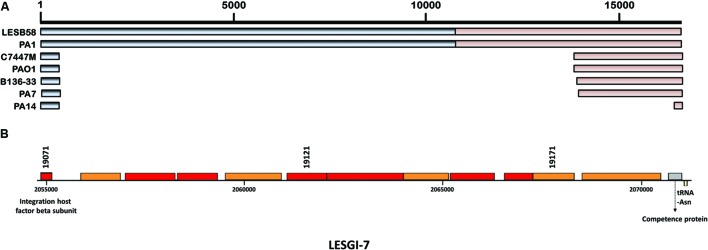
**Segmental and gene map of LESGI-7 (2054683-2071280 bp). (A)** LESGI-7 in *P. aeruginosa* LESB58 compared against the homologs in other *P. aeruginosa* strains using BLASTN. Segments LESGI-7-C1 and LESGI-7-C2, shown in shades of black and brown, respectively, differ in oligonucleotide composition **(B)** Genes on LESGI-7: Integration host factor beta subunit and a flanking tRNA gene, indicative of the horizontal acquisition, are indicated.

It is a cluster of 14 genes involved in determining the lipopolysaccharide (LPS) O-antigen serotype. This region is characterized by a high plasticity. Twenty different O-antigen serotypes of *P. aeruginosa* have been characterized (Liu and Wang, 1990). The O-antigen genes vary among the strains of *P. aeruginosa*. Sequence comparison showed that similar sequences were also present in PA1 strain and partially in C7447M, PAO1, B136-33, and PA7 (**Figure 5A**).

LESGI-7 (**Figure 5B**) is also referred to as a replacement island, i.e., an island arising from diversifying selection (Smith et al., 2005; Kung et al., 2010). LESGI-7 encoded proteins function in chemical modification of sugars and their assembly into polysaccharide subunits. These subunits are ligated to form polysaccharide chain of O antigen (Rocchetta et al., 1999). LPS, which forms major component of cell wall, has been widely studied because of its role in attachment (Makin and Beveridge, 1996), evasion from host defenses (Hancock et al., 1983; Dasgupta et al., 1994) and establishment of the infection (Tang et al., 1996). Compositional atypicality, genetic instability, presence of integration host factor beta subunit and a flanking tRNA-Asn gene indicate the likely horizontal acquisition of this island.

The LPS O-antigen-serotype genes are well distributed in different strains of *P. aeruginosa* (**Table 1**). GEMINI identified this island as composed of two compositionally distinct segments suggesting at least two transfer events at this locus. Consistent with our predictions, both LESGI-7-C1 (left segment- shown in gray in **Figure 5A**; genes PALES_19071- PALES_19141, 50.7% GC content) and LESGI-7-C2 (right segment- shown in light brown in **Figure 5A**; genes PALES_19151- PALES_19191, 60.46% GC content) show significant difference in GC content. Both LESGI-7-C1 and LESGI-7-C2 carry genes encoding O-antigen serotype O6. LESGI-7-C2 also carries a competence protein (PALES_19191). It has high similarity to ComEA protein of *P. aeruginosa* (BLAST-100% query coverage and 99% identities). ComEA plays an important role in natural transformation (Seitz et al., 2014). High variability in this region (Kung et al., 2010) makes it difficult to ascertain the putative donors of these segments. We discuss about the potential origin of the two segments in “Analysis of mosaic genomic islands” section below.

### LESGI-8

It corresponds to the island near 2.6-Mbp region in the unverified island region (UVIR; **Figure 1**). As noted earlier, MJSD (Arvey et al., 2009), SIGI- HMM (Waack et al., 2006), and IVOM (Vernikos and Parkhill, 2006) detected only parts of this island. This island is also present in B136-33, C7447M, PAO1, and SCV20265 strains. Homology search shows the presence of parts of this island in PA1 and PA7 (Supplementary Figure S2A).

LESGI-8 is composed of 13 genes, and encodes a probable type II secretion system (T2SS; Supplementary Figure S2B). T2SS, like other bacterial secretion systems, plays an important role in pathogenesis and virulence (Bleves et al., 2010). In the *P. aeruginosa* core genome, T2SS is encoded by a set of 11 contiguous genes arranged in two operons and an additional gene located outside of the two operons (Bleves et al., 2010). Of these T2SS genes, six were found on this island and the rest in the core genome. These include general secretion pathway proteins GspE (PALES_24271), GspF (PALES_24281), GspG (PALES_24291, PALES_24301), GspI (PALES_24311), and GspJ (PALES_24321). GspE acts as ATPase and GspF, GspG, GspI, and GspJ are integral membrane proteins (Filloux, 2004; Douzi et al., 2012).

All the genes found on this island are well distributed in the *P. aeruginosa* group but do not have significant homology outside of the *P. aeruginosa* group (NCBI taxid: 136841; **Table 1**), suggesting an ancient transfer of this island into the genome of the common ancestor of *P. aeruginosa* clade.

### LESGI-9

Similar to LESGI-8, it was identified near ~2.65 Mb region in the UVIR (**Figure 1**). This island was identified by MJSD as well (Arvey et al., 2009). LESGI-9 was almost entirely found in B136-33, C7447M, PA1, PA7, PAO1, SCV20265, and PA14 strains (Supplementary Figure S3A). It was absent from other strains included in this study.

This island consists of a partial gene cluster of NADH dehydrogenase, methyltransferase, an uncharacterized protein and an exported protein (Supplementary Figure S3B). Gene *PALES_24541* on this island, which codes for an uncharacterized protein, was absent in all other strains analyzed in this study.

### LESGI-10

**FIGURE 6:**
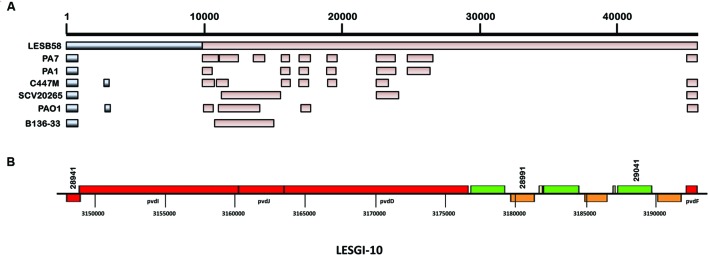
**Segmental and gene map of LESGI-10 (3147091-3192857 bp). (A)** LESGI-10 in *P. aeruginosa* LESB58 compared against the homologs in other *P. aeruginosa* strains using BLASTN. Segments LESGI-10-C1 and LESGI-10-C2, shown in shades of black and brown, respectively, differ in oligonucleotide composition. **(B)** Genes on LESGI-10: This replacement island harbors a cluster of pyoverdine genes (pvdI to pvdF).

This is another replacement island harboring genes involved in pyoverdine synthesis (Smith et al., 2005). This region is known to acquire frequent mutations (Smith et al., 2005). Therefore only parts of this island are identified in other strains of *P. aeruginosa* by the sequence comparison (**Figure 6A**). The genes of this island were classified into two distinct clusters by GEMINI. One cluster is comprised of *sip* and *pvdI* genes and the other cluster contains all the remaining pyoverdine genes from *pcdJ-pvdF* (**Figure 6B**).

Pyoverdines are primary siderophores of *P. aeruginosa*, which are important for iron acquisition and may also play a role in virulence (Meyer et al., 1996; Takase et al., 2000). LESB58 has a Type III pyoverdine region. The Type III pyoverdine region is characterized by unusual codon and oligonucleotide usages, indicating perhaps a recent history of HGT in the evolution of LESGI-10 (Mahajan-Miklos et al., 1999). Genes in the pyoverdine region showed high similarity to the genes in the soil bacteria, mainly, *Azotobacter vinelandii* and *Agrobacterium tumefaciens* (Smith et al., 2005).

### LESGI-11

It is a mosaic island with a bipartite structure (Supplementary Figure S4A). Segment LESGI-11A is absent, almost in its entirety, in all strains of *P. aeruginosa*. However, the segment LESGI-11B was almost completely present in PAO1 and PA7, and partially in C7447M, B136-33, SCV20265, and PA7 strains.

The segment LESGI-11A consists of six genes, all of which code for hypothetical proteins. The second segment LESGI-11B is comprised of 34 genes including an incomplete cluster of phenazine genes, from *phzG2–phzC2*, involved in phenazine biosynthesis (Supplementary Figure S4B). Phenazines are metabolites that function in virulence (Mahajan-Miklos et al., 1999) and microbial competitiveness (Mazzola et al., 1992).

### LESGI-12

LESGI-12 was identified completely in B136-33, C7447M, PA1, PA7, PAO1, SCV20265 but was absent in the other strains (Supplementary Figure S5A). It harbors eight genes including those that code for membrane proteins (CmpX and OprF), metal ion transporter (PALES_35561) and a regulatory gene (PALES_35531; Brinkman et al., 1999). The *oprF* gene (Supplementary Figure S5B) codes for a structural outer membrane porin involved in maintaining the cell shape and the ability to grow in low osmolarity medium (Brinkman et al., 1999). The presence of OprF contributes to increasing antibiotic resistance by making the *P. aeruginosa* less permeable (Hancock and Speert, 2000; Peng et al., 2005). The expression of *oprF* is elevated in Australian epidemic strain of *P. aeruginosa* compared to non-transmissible strains PAO1 and PA14, perhaps indicating its role in transmissibility (Hare et al., 2012). The genes harbored on this island have homologs in several strains of *P. aeruginosa*, perhaps suggesting an ancient transfer or multiple acquisitions in the *P. aeruginosa* strains. The nearest, non-*Pseudomonas* homolog of OprF is found in *Azotobacter chroococcum* and *Azotobacter vinelandii* with 72 and 69% identity, respectively. *A. chroococcum* was isolated in 1901 as an infectious agent associated with tobacco-mosaic disease, and it fixes nitrogen under aerobic conditions (Shivprasad and Page, 1989). It is possible that *P. aeruginosa* acquired *oprF* gene and ameliorated to exploit its codon usage as the presence of this conferred many selective advantages.

### LESGI-13

Sequence comparison showed that region similar to LESGI-13 is also present in C7447M, PA1, PAO1, SCV20265, B136-33, and in-part in PA7 and PA14 (Supplementary Figure S6A). It contains chaperone-usher pathway (*cup*) and *tolQRAB* gene clusters (Supplementary Figure S6B). The *cup* gene cluster is involved in assembling fimbriae (Ruer et al., 2007). These fimbrial structures have been reported to play a role in pathogenesis (Hull et al., 1981) and biofilm formation (Marklund et al., 1992; Soto and Hultgren, 1999). In particular, the *cupC* system found on this island is known to be involved in biofilm formation (Vallet et al., 2001; Ruer et al., 2007).

The *tolQRAB* cluster was originally identified in *P. aeruginosa* (Dennis et al., 1996) and was shown to be involved in the uptake of pyocin AR41 (Dennis et al., 1996). The *E. coli tol* system has been shown to contribute to the integrity of cell membrane, resistance to antibiotics and detergents, and colicin uptake (Lazzaroni and Portalier, 1981; Lazzaroni et al., 1989; Webster, 1991). These genes are required for the uptake of *V. cholerae* CTXφ, a lysogenic filamentous bacteriophage that encodes cholera toxin (Heilpern and Waldor, 2000).

### LESGI-14

It was found almost entirely in PA1 and B136-33 strains and partly in C7447M, PAO1, SCV20265, PA7, and PA14 (Supplementary Figure S7A). However, the individual genes harbored by this island are well distributed in the *P. aeruginosa* strains (**Table 1**). It contains genes coding for mostly hypothetical proteins and its role needs to be further investigated (Supplementary Figure S7B).

### LESGI-15

The complete sequence of LESGI-15 was found in B136-33, C7447M, PA1, PA7, PAO1, and SCV20265 strains (Supplementary Figure S8A). The genes on this island are well spread amongst the strains of *P. aeruginosa* (**Table 1**). It houses a TonB-dependant receptor gene, which is known to be involved in iron uptake in *P. aeruginosa* (Poole et al., 1996). It also harbors a gene encoding a putative virulence-associated protein (PALES_50601; Supplementary Figure S8B).

### LESGI-16

This island was also found in B136-33, C7447M, PA1, PA7, PAO1, and SCV20265 strains while absent from the other strains (Supplementary Figure S9A). Phyletic pattern again indicates a likely ancient transfer of LESGI-16 (**Table 1**). It harbors genes coding for synthase, mutase, isomerase, reductase and nucleotidyltransferase, and tRNA genes (Supplementary Figure S9B).

### LESGI-17

**FIGURE 7:**
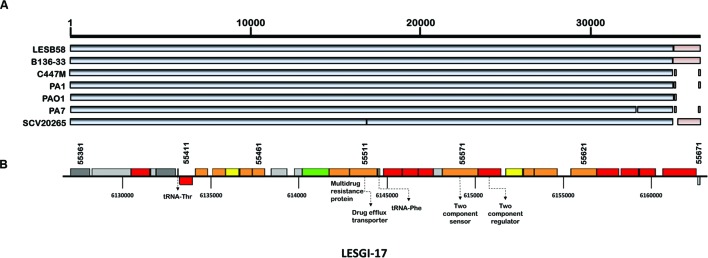
**Segmental and gene map of LESGI-17 (6127068-6164062 bp). (A)** LESGI-17 in *P. aeruginosa* LESB58 compared against the homologs in other *P. aeruginosa* strains using BLASTN. Segments LESGI-17-C1 and LESGI-17-C2, shown in shades of black and brown, respectively, differ in oligonucleotide composition. **(B)** Genes on LESGI-17: The presence of genes encoding a multidrug resistance protein and a drug eﬄux transporter is indicated.

Sequence comparison identified this island to be present almost in entirety in B136-33 and partly in C7447m, PA1, PAO1, PA7, and SCV20265 strains (**Figure 7A**). LESGI-17 (**Figure 7B**) is a cluster of 33 genes including genes encoding multidrug resistance proteins, drug eﬄux transporters and probable two component regulatory system, and tRNA genes. This island is mosaic, with one segment containing a single gene, *PALES_55661*, coding for a hypothetical protein and the other segment containing the rest of the LESGI-17 genes.

This island is present in the close relatives of LESB58 (**Table 1**), indicating an ancient transfer, however, its atypical composition as indicated by GEMINI suggests that this island has not yet completely ameliorated its composition to that of the native genome, as was observed with several other LESGIs.

The GI features identified in these novel islands are summarized in Supplementary Table S2.

### Missed GIs

Islands identified by Winstanley et al. (2009) but missed by GEMINI were earlier annotated as Prophage 1 that codes for pyocin R2, Prophage 4, and LESGI-2 (VI-3; **Table 1**). The lack of atypical compositional biases (Winstanley et al., 2009) precluded the detection of these islands by our method. This suggests that these islands could perhaps be one of the early acquisitions resulting in the loss of compositional bias.

### Analysis of Mosaic Genomic Islands

**FIGURE 8:**
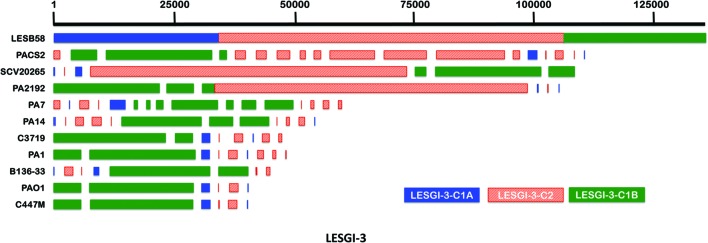
**Genomic island LESGI-3 (2798951-2935111 bp).** Mosaic compositional structure of LESGI-3, compared against homologs in ten other *P. aeruginosa* strains using BLASTN. Segments LESGI-3-C1A and LESGI-3-C1B, shown in blue and green, respectively, share similar composition.

**FIGURE 9:**
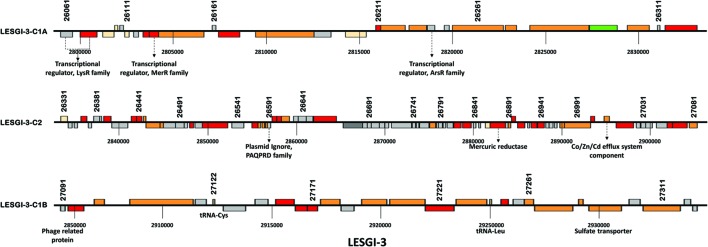
**Gene map of LESGI-3: Genes harbored by individual segments of LESGI-3 (2798951-2935111 bp).** LESGI-3-C1A carries transcriptional regulator genes. LESGI-3-C2 carries metal resistance genes encoding mercuric reductase and Co/Zn/Cd eﬄux system component. LESGI-3-C1B harbors a phage gene and tRNA genes.

Mosaic GIs have previously been described in *P. aeruginosa* (Klockgether et al., 2004; Mathee et al., 2008). Analysis of the island-rich region in LESB58 strain demonstrated the ability of GEMINI to decipher the mosaic structure of the GIs (**Figure 1**). Among the verified islands, VI-2 and VI-4 showed the mosaic structure (**Figure 1**). This study identified six mosaic islands in LESB58 using GEMINI (LESGI-3, 7, 10, 11, 17, and Prophage-5; **Table 1**; **Figures 1**, **3**, **5**, **6**, **8**, and **9**; Supplementary Figures S4 and S7), of which three are novel (LESGI-7, 10, and 11). Overall, deciphering the mosaic structure through recursive segmentation and agglomerative clustering helped not just identify the islands in *P. aeruginosa* LESB58 more precisely but also reveal the underlying structural organization of the acquired genetic elements in this strain (**Figure 3**; **Table 1**). Our sensitive and robust detection of islands and their underlying structures provided an opportunity to examine their distributions in closely related genomes and trace their ancestries. Comparative genomics of these islands shed light on the disparate lineages of the segments comprising the mosaic GIs and their contributions to pathogenicity.

LESGI-3 is among the 11 annotated islands and corresponds to the fourth verified island (VI-4) in **Figure 1**. The three segments that compose this island in *P. aeruginosa* LESB58, annotated LESGI-3-C1A, LESGI-3-C2, and LESGI-3-C1B (LESGI-3-C1A and LESGI-3-C1B share similar composition) displayed unusual phyletic pattern in their distributions in the closely related strains that included rearrangements, inversions, and losses of DNA segments (**Figure 8**). This island in LESB58 appears unique, as none of the other strains carries a GI with similar structure, and therefore is a potential biomarker candidate for this strain. Segment LESGI-3-C1A in particular was almost entirely absent in the strains used in this study. Besides harboring genes conferring metal resistance (PALES_26101, PALES_26181, PALES_26231, and PALES_26321), it also carries genes encoding transcriptional regulators (PALES_26061, PALES_26071, PALES_26141, and PALES_26241). None of these transcriptional regulators is present in the other representative strains. The LysR type transcriptional regulator has previously been shown to regulate genes involved in pathogenesis (Cao et al., 2001; Maddocks and Oyston, 2008). Both *PALES_26061* and *PALES_26071* harbored on LESGI-3-C1B have been annotated as members of LysR type transcriptional regulator gene family, whose role is not yet well-understood (**Figure 9**).

If GEMINI deciphers the mosaic structure of GIs robustly, then our hypothesis is that the segments within our predicted islands have distinct evolutionary origins, and therefore, the segments should display high BLAST coverage and identity to different distant taxa. We indeed observed so. The first segment, LESGI-3-C1A (**Figures 8** and **9**), showed the highest similarity to *Acidovorax ebreus* TPSY (query coverage 92% and nucleotide identity 99%) belonging to beta-proteobacteria, whereas *P. aeruginosa* is a gamma-proteobacterium. *Acidovorax ebreus* is an anaerobic nitrate-dependent Fe (II) oxidizer. The second segment, LESGI-3-C2, showed maximum similarity with another beta-proteobacterium *Achromobacter xylosoxidans* (query coverage 91% and nucleotide identity 97%). The third segment, LESGI-3-C1B, didn’t display significant similarity with any distant taxa (the closest one had only 9% query coverage), suggesting that this segment has arrived from an organism whose genome is yet to be sequenced or has undergone significant changes since its acquisition.

The high nucleotide-level similarity between the segments of LESGI-3 and potential donors suggests that LESGI-3 is a recent acquisition. Donors of ancient acquisitions could be difficult to predict because of the amelioration or divergence of a sequence since its acquisition. In instances where we didn’t observe significant nucleotide identities, we went further to assess conservation at the amino acid level using the BLASTX program. Furthermore, we analyzed the distribution of GI-borne genes at different taxonomic levels (Supplementary Table S3). For example, if a gene is present in the members of the *Pseudomonadaceae* family, however, is either absent or sparsely distributed outside of the family but within the order *Pseudomonadales*, then we considered it as a putative alien gene.

LESGI-7 and LESGI-10 are replacement islands with high plasticity (Smith et al., 2005). This makes finding the potential donors of the component segments difficult. Frequent genomic rearrangements may further aggravate this problem. The gene-by-gene analysis of the segments suggests several potential donors for LESGI-7A (PALES_19071–PALES_19141), including *Streptococcus pneumoniae, Microbulbifer variabilis, Thauera* sp. MZ1T*, Vibrio Cholerae, Nitrosococcus oceani, Desulfotomaculum kuznetsovii, Desulfotaela psychrophila*, and *Burkholderia* sp. SJ98. Unusually high similarity to genes in distant lineages, outside of *Pseudomonadales* than within this order (excluding *Pseudomonadaceae*), as elicited by the BLAST best hits, indicated the likely origins of the LESGI-7A genes (Supplementary Table S3). Similarly, the LESGI-7B genes had the best BLAST hits outside of the *Pseudomonadales* order, to organisms such as *Bordetella petrii, Polaromonas naphthalenivorans*, *Microbulbifer variabilis, Gamma proteobacterium* L18, and *Gallibacterium genome* sp. 2 (Supplementary Table S3).

The potential donors of the two genes on LESGI-10A, PALES_28941 and PALES_28951, based on the sequence comparison, could be *gamma proteobacterium* L18 and *Archangium gephyra*, respectively, again from a different taxonomic order (Supplementary Table S3). The potential donor for LESGI-10B is inferred to be either *Cellvibrio japonicus*, *Azospirillum thiophilum* or one of the members belonging to *Burkholderiales* order, namely, *Alcaligenes faecalis, Janthinobacterium* sp. HH01 or *Burkholderia* sp. (Supplementary Table S3).

LESGI-11 and LESGI-17 also showed similar atypical distribution of their genes. These genes while conserved within *Pseudomonadaceae* were either sparsely distributed or absent within other families of the order *Pseudomonadales* but again well-conserved in certain lineages outside of this order. LESGI-11A consists of a group of hypothetical proteins. Genes on this segment have aberrant phylogenetic distribution within the *P. aeruginosa* group. The organisms with top BLAST hits for these genes included *Xanthomonas arboricola, Vibrio cholerae, Spongiibacter* sp.*, Poephila acuticauda, Aquitalea magnusonii*, and *Yersinia pseudotuberculosis* (Supplementary Table S3). We found many of these LESB58 genes missing from several strains of *P. aeruginosa* indicating either a recent acquisition or gene loss from multiple strains. Conversely, LESGI-11B may likely be an ancient acquisition, as evinced by its ubiquity within *Pseudomonadaceae* family. *Streptococcus pneumoniae* was found to be among the best hits for nine out of the 33 LESGI-11B genes (Supplementary Table S3).

Of the 31 genes on LESGI-17A, nine genes had the best hits in *Streptococcus pneumoniae*. Sequence comparison indicates other potential donors of the segment to be *gamma proteobacterium* L18 or *Burkholderia* sp. LESGI-17B is comprised of two genes, a beta-ketoacyl gene with the best hit in *Paraglaciecola arctica* and a hypothetical protein with the best hit in *Fusicatenibacter saccharivorans* (Supplementary Table S3).

We further examined whether these best BLAST hits in distant taxa originate from either the core or the accessory. In all instances, these genes were found to originate from core genomes of the donors (Supplementary Table S4)

### Functional Classification of Core and Accessory Genomes

The above analyses demonstrated the sensitivity and robustness of GEMINI in identifying the GIs. This motivated the functional analyses of the native or core and the accessory genome of the LESB58 strain. Core genome has traditionally been defined based on sequence comparisons and ortholog analyses (Spencer et al., 2003; Lee et al., 2006; Cramer et al., 2011). As expected, as more and more new strains are identified and sequenced, the core genome keeps shrinking (Klockgether et al., 2011). In contrast, our predicted core genome is comprised of segments with compositional biases representing the mutational proclivities of native or ancestral genes. By definition, this core genome contains the typical (native) genes present in the genome of interest, which may or may not have been retained in other strains of *P. aeruginosa*. This allowed identifying the core genome of *P. aeruginosa* LESB58 based on oligonucleotide composition, independent of sequence comparison across *P. aeruginosa* strains. Accordingly, the *P. aeruginosa* LESB58 core genome is defined here as the collection of genes bearing the oligonucleotide compositional signature of ancestral genes resident within the largest cluster generated by GEMINI. As mentioned, the recursive segmentation and agglomerative clustering were performed within the framework of a second order Markov chain model and therefore, the core of the genome was identified based on trinucleotide compositional signature. The largest cluster, comprised of ~87.2% of the genome, represents the core genome of *P. aeruginosa* LESB58 (shown in gray in **Figure 3**).

**FIGURE 10:**
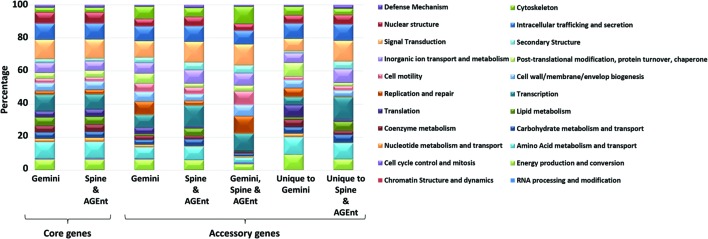
**Functional distributions of *P. aeruginosa* LESB58 genes.** The distribution of genes identified either core or accessory by GEMINI, & Spine and AGEnt (Ozer et al., 2014).

To assess the functions of core genes, we obtained the functional classification data from *Pseudomonas* Genome Database^2^ (Winsor et al., 2009, 2011). The core genomes predicted by GEMINI, and Spine and AGEnt (Ozer et al., 2014) did not show significant difference in their functional composition (**Figure 10**; Supplementary Figure S10). GEMINI classified ~16% core genome as involved in information storage and processing, ~23% dedicated for cellular processes and signaling, ~39% with metabolic functions, and the remaining (~22%) as yet uncharacterized or poorly characterized, similar to the prediction by Spine and AGEnt (Supplementary Figure S10).

Of the 698 alien genes identified by GEMINI, 465 were shared with the Spine and AGEnt output of 1403 accessory genes. The overlapping set of 465 accessory genes (two-third of the GEMINI predicted alien genes) thus represents accessory genome with a high confidence. The remaining 233 were classified as core genes by Spine and AGEnt, due to their presence in most *P. aeruginosa* strains. However, their atypical composition and physical association with other atypical genes within the genome suggest their likely lateral acquisition in the genomes of many *P. aeruginosa* strains or in the genome of a common ancestor. We have thus included these 233 genes as part of accessory genome.

We also analyzed the functional classification of the accessory genes that were predicted by GEMINI but not by Spine and AGEnt, and vice-versa (**Figure 10**). Accessory genes unique to GEMINI were enriched for functions, such as, energy production and conversion, amino acid metabolism and transport, nucleotide metabolism and transport, coenzyme metabolism, translation, replication repair, cell wall/membrane/envelop biogenesis, post-translational modification, protein-turnover, chaperone, and cytoskeleton (**Figure 10**). Notably, previous studies have also reported HGT of translation, transcription, replication, recombination, and repair-associated genes (Valot et al., 2015; Zhaxybayeva and Gogarten, 2002). Likewise, the accessory genes predicted by Spine and AGEnt but not by GEMINI are enriched in RNA processing and modification, cell cycle control and mitosis, carbohydrate metabolism and transport, lipid metabolism, transcription, cell motility, inorganic ion transport and metabolism, secondary structure, signal transduction, intracellular trafficking and secretion, nuclear structure, and defense mechanism (**Figure 10**).

## Discussion

A previously published method (Azad and Li, 2013) augmented by segment context information was used to decipher novel genomic elements that may be contributing toward hyper-virulence and antibiotic resistance in bacterial pathogens. Here, we used this modified method to revisit *P. aeruginosa* LESB58 whose epidemic nature remains an enigma. Functional analyses of the putative GIs indicate the propensity of LESB58 to acquire GIs that help increase its fitness by providing new functions. This study reveals that by acquiring numerous GIs LESB58 has acquired additional virulence (LESGI-6, 8, 10, 11, 13, and 15), drug and metal resistance (LESGI-12 and -17), adapted to the host environment by evading immune response (LESGI-7), and added versatility to its metabolic repertoire (LESGI-9, -16, and -17). Furthermore, GEMINI shed light on mosaic structures of GIs and their potential to contribute to the evolution of pathogenicity.

The robustness of GEMINI stems from its ability to identify even weakly atypical genomic segments. Furthermore, the top-down genome segmentation allows to delineate the boundaries of GIs more precisely than the frequently used moving window methods. Our analyses also highlight the problems associated with alignment-based approach to identify large regions with atypical composition. Our composition-based approach, however, is able to not just delineate the GIs but also deconstruct their mosaic structure. Interestingly, our data suggest ancient acquisition for many of the predicted mosaic GIs, which may otherwise be difficult to ascertain using methods that examine gene distribution in close relatives. Many predicted GIs had a significant number of genes that were present in a majority of the *P. aeruginosa* strains (**Table 1**). While we used a simple majority rule to infer conserved genes in the *P. aeruginosa* strains, this is a more relaxed estimate of the conserved genes, and it is possible many of these conserved, predicted island borne genes could indeed be alien genes which might have got transmitted to multiple strains since their acquisition, or might have been acquired independently by multiple strains. Furthermore, it is possible that the islands displaying conserved pattern among the strains might have been acquired by the common ancestor of *P. aeruginosa*. Ancient acquisitions are also difficult to detect using composition-based methods, however, the ability of GEMINI to simultaneously analyze multiple genes via recursive segmentation enabled detection of these weakly atypical regions in the LESB58 strain. These loci in LESB58 may have been subject to recurrent evolutionary changes, including gain and loss of genes, over a longer period. This was revealed in this study, with segments of several mosaic GIs seem to have acquired genes from different lineages. These hotspots of gene acquisition may have played a major role in the emergence and evolution of pathogenic and resistant strains in this clade.

Notably, unlike alignment-based methods, including BLAST, our compositional approach is not sensitive to evolutionary changes such as rearrangements that disrupt the genomic contiguities confounding the ability to discern shared evolutionary signals. Analysis of the segments comprising LESGI-3 indeed supports this– the segment LESGI-3-C1B lacks a significant BLAST hit, however, our study shows that LESGI-3-C1A and LESGI-3-C1B (**Figure 8**) share significant compositional similarity, suggesting that the donors of these DNA elements likely belong to the same phylogenetic taxa. In addition, it provides a window into the ecological niche that was shared by these three species. In fact, both *Acidovorax* and *Achromobacter* species have been found in the lungs CF patients (Jakobsen et al., 2013; Trancassini et al., 2014). The latter is considered an emerging pathogen in various clinical settings, such as pneumonia, catheter-associated infections and urinary tract infections (Palacios-Gómez et al., 2014).

The genomes of *Acidovorax ebreus* and *Achromobacter xylosoxidans* were recently sequenced (Byrne-Bailey et al., 2010; Jakobsen et al., 2013). Both species code for genes that can confer antibiotic resistance and other virulence phenotypes. For an example, it is a huge concern that *Acidovorax ebreus* has regions that may confer resistance to lead (*pbrRATARTBC*), arsenate (*arsRDAB*), and mercury (*merRPCADE*; Byrne-Bailey et al., 2010). It has been demonstrated that metal resistance gives rise to concomitant antibiotic resistance (Alonso et al., 2001; Baker-Austin et al., 2006). In addition, *Acidovorax ebreus* harbors one CRISPR (clustered, regularly interspaced, short palindromic repeats) region (Barrangou et al., 2007) that has been used as a biomarker for *P. aeruginosa* epidemic strains. Together, these data suggest gene flow between *P. aeruginosa* and *A. ebreus*.

Availability of verified islands in the LESB58 strain afforded the opportunity to assess the strengths and weaknesses of GI detection methods including GEMINI. While GEMINI performed well in localizing large validated islands, it completely missed a relatively smaller validated island, VI-3 (**Figure 1**), which was, however, picked by IslandViewer and Zisland Explorer albeit as a part of their largest predictions. This island was also missed by other methods tested here. Failure of these tools to identify this verified island can be attributed to the lack of compositional bias or other identifying features (Langille et al., 2010). It is possible that this island could be representing an ancient transfer event and therefore the composition of VI-3 could have ameliorated to the recipient genome composition over the passage of time since this transfer (Langille et al., 2010). Alternatively, it may have been acquired from a phylogenetically proximal donor. This is reflected in its sequence composition similar to that of the native genome as indicated by its assignment to the native cluster by GEMINI, and as previously reported (Winstanley et al., 2009). In addition, V1-3 is present in many *P. aeruginosa* strains (Langille et al., 2010). Our results highlight the complementary strengths of different methods and therefore the need to exploit the complementarity to take GI detection to new heights. Future work could focus on more robust evaluation of GI detection methods including GEMINI across diverse species and leverage this information to develop integrative approaches for more robust detection of GIs.

While the focus of this study was to decipher the accessory genome of LESB58, it also afforded an opportunity to examine the native or core genome predicted by GEMINI. Previous studies using comparative genomics have reported ~90% of the LESB58 genome as being the part of *P. aeruginosa* core genome (Spencer et al., 2003; Tümmler, 2006; Mathee et al., 2008). Comparative analysis of 18 *P. aeruginosa* genomes by Spine and AGEnt (Ozer et al., 2014) identified ~75.1% of the LESB58 genome as the core genome (Ozer et al., 2014), which shrank to ~74.3% when 22 strains were used. Spine and AGEnt identified a significantly smaller core genome compared to GEMINI, which classified ~87.2% of the LESB58 genome as core. However, sequence comparison methods for core genome determination, such as Spine and AGEnt, are prone to eliminating the ancestral genes lost from one or more strains.

Compared to Spine and AGEnt, the composition-based approaches offer the advantage of determining the genome specific core, independent of genome comparison, and thus present a “stable” core, which has otherwise been shrinking with the addition of more sequenced genomes. Furthermore, the genome segmentation can also help identify ancestral non-coding sequences, such as, those of non-coding RNA genes.

## Conclusion

The epidemic strain LESB58 remains enigmatic despite significant advances in the pathogenomics of *P. aeruginosa*. Here we have shown that the difficulty in understanding the emergence of hyper-virulent strains, such as LESB58, lies partly in the inability of the current approaches in localizing large structures encoding virulence and/or resistance functions and in deconstructing the mosaic structures of GIs that often have direct bearing on the increased virulence observed in newly emerged pathogenic strains. Not unexpectedly, we identified many new GIs through the LESB58 genome segmentation, most of which harbored genetic elements encoding antibiotic resistance and virulence factors. Furthermore, the same approach shed light on the mosaics that a number of these islands are, and how genome innovation driven by HGT results in unique genetic markers that characterize pathogenic strains and their traits. Comparative genomics revealed that many of these islands are mosaics of DNAs of opportunistic pathogens co-residing within the lungs of CF patients. This suggests that the source of the new traits could be hybrid DNAs created via recombination between co-resident pathogens in CF patients. Apparently, this frequent DNA exchange is driven by evolutionary pressures on bacteria to survive in hostile environments such as the lungs of CF patients. Our study also identified a potential biomarker for the LES strains. Future studies should focus on such genetic markers conferring complex traits, whose inactivation or expulsion could diminish the virulence or resistance potentials of new or emerging pathogenic strains.

## Author Contributions

RA conceived and designed the study. MJ and RA performed the experiments and analyses, and wrote the manuscript. MJ, KM, and RA participated in critical analysis and interpretation of the data. KM and RA critically reviewed and edited the manuscript. MJ, KM, and RA read and approved the final manuscript.

## Conflict of Interest Statement

The authors declare that the research was conducted in the absence of any commercial or financial relationships that could be construed as a potential conflict of interest.

## Footnotes

1 http://www.ncbi.nlm.nih.gov/bioproject/185969

2 http://www.pseudomonas.com/
