# Supplementary material for: Identification of Novel Genomic Islands in Liverpool Epidemic Strain of Pseudomonas aeruginosa Using Segmentation and Clustering
Source: Front Microbiol. 2016 Aug 3;7:1210. doi: 10.3389/fmicb.2016.01210 (PMC4971588; doi:10.3389/fmicb.2016.01210)
Supplement: Supplementary file 5 [file Presentation_1.PPTX]

## Slide 1
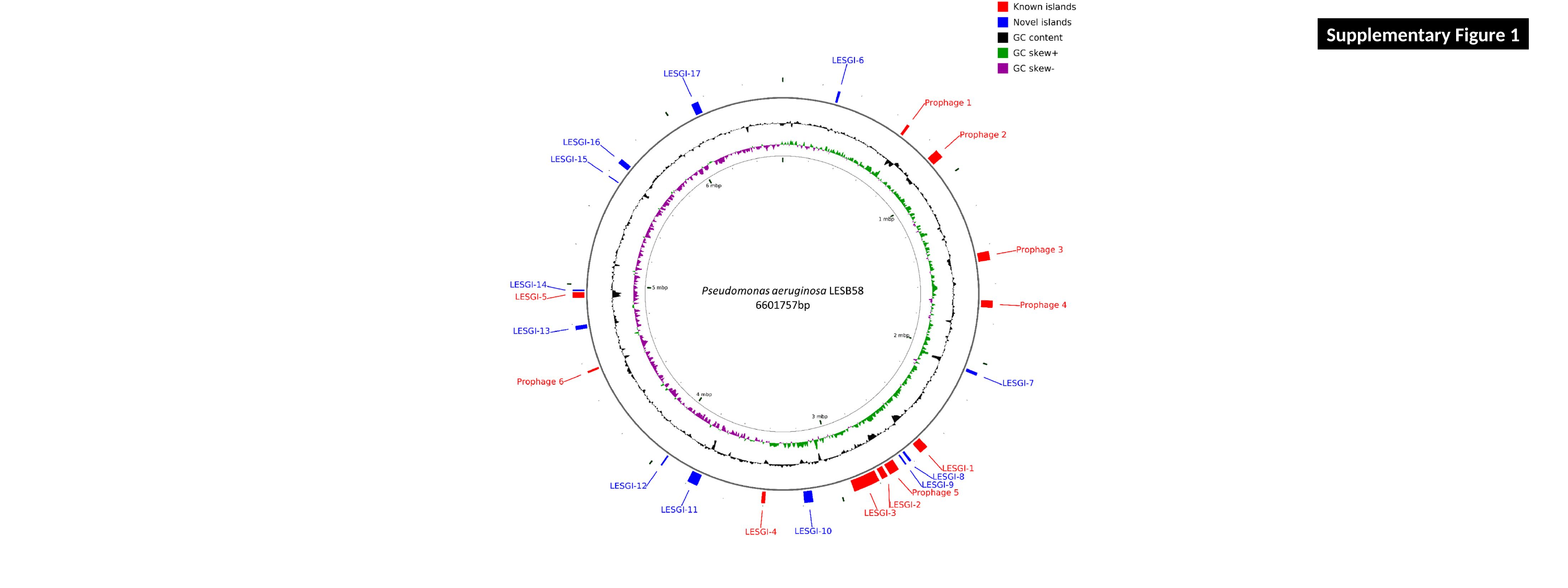

Supplementary Figure 1

## Slide 2
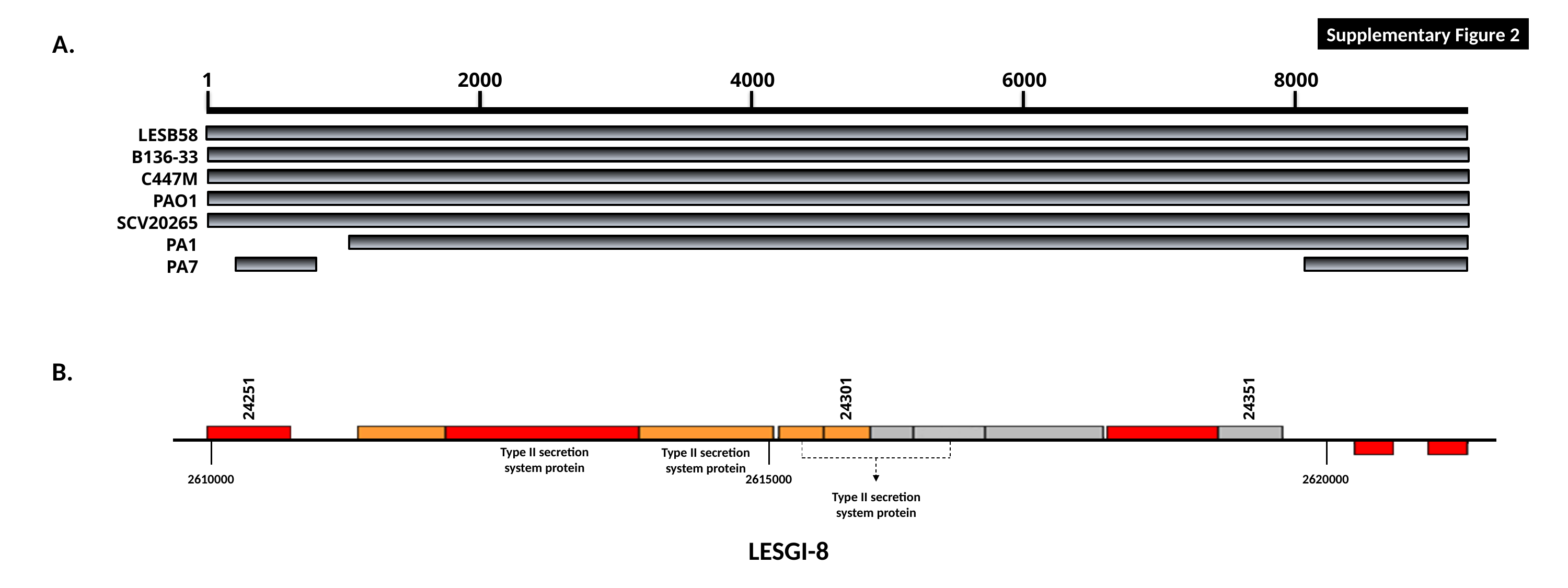

Supplementary Figure 2
A.
1
2000
4000
6000
8000
LESB58
B136-33
C447M
PAO1
SCV20265
PA1
PA7
24251
24301
24351
Type II secretion system protein
Type II secretion system protein
2610000
2615000
2620000
Type II secretion system protein
B.
LESGI-8

## Slide 3
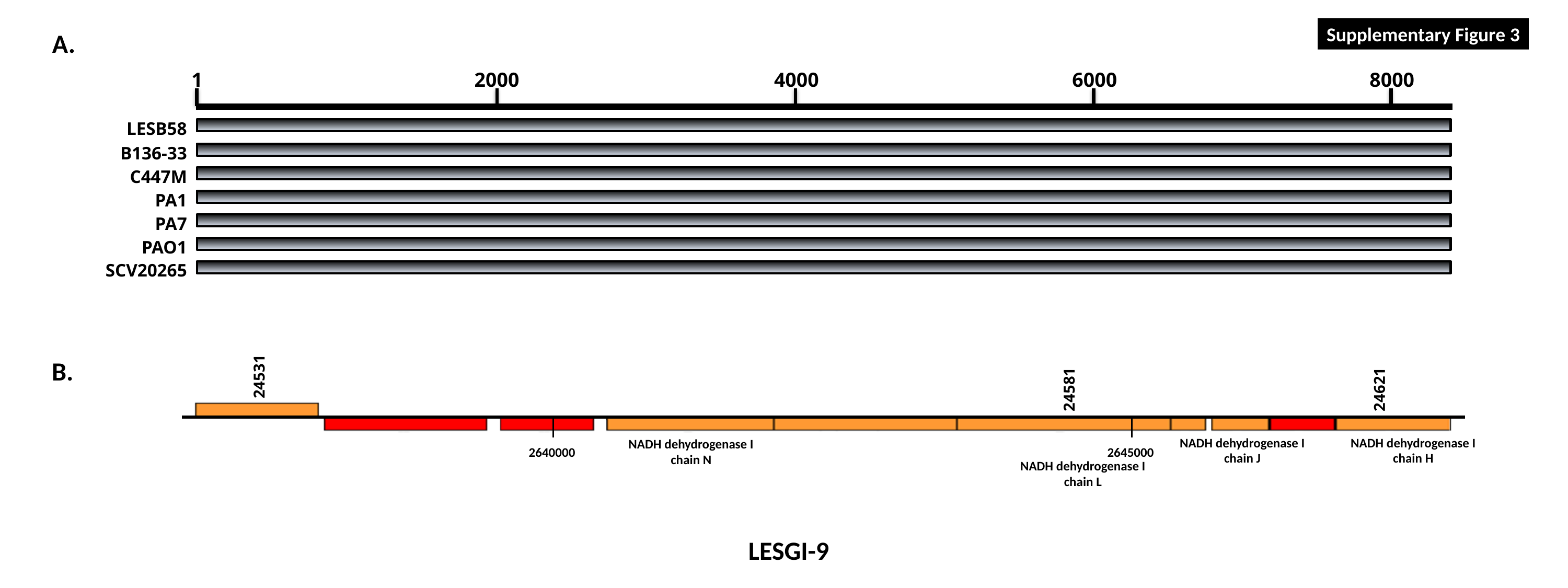

Supplementary Figure 3
A.
1
2000
4000
6000
8000
LESB58
B136-33
C447M
PA1
PA7
PAO1
SCV20265
24531
24581
24621
NADH dehydrogenase I chain J
NADH dehydrogenase I chain H
NADH dehydrogenase I chain N
2640000
2645000
NADH dehydrogenase I chain L
B.
LESGI-9

## Slide 4
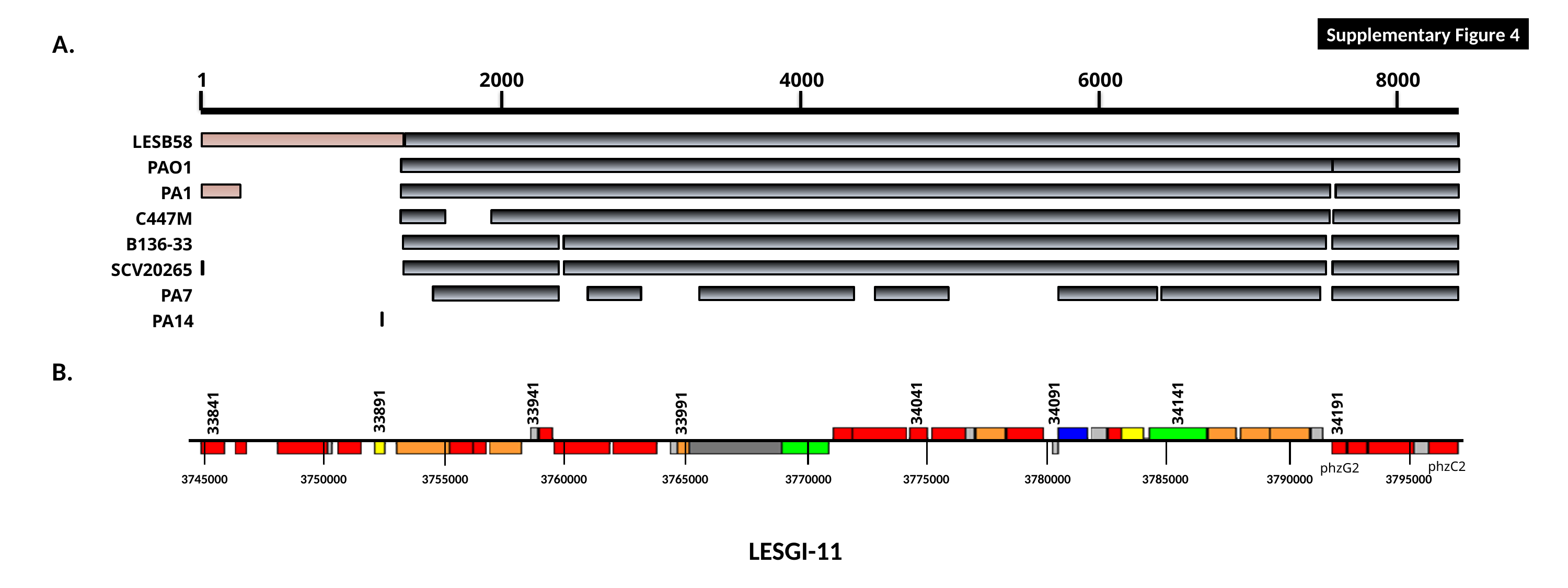

Supplementary Figure 4
A.
1
2000
4000
6000
8000
LESB58
PAO1
PA1
C447M
B136-33
SCV20265
PA7
PA14
33941
34041
34091
34141
33891
33841
33991
34191
3745000
3750000
3755000
3760000
3765000
3770000
3775000
3780000
3785000
3790000
3795000
phzC2
phzG2
B.
LESGI-11

## Slide 5
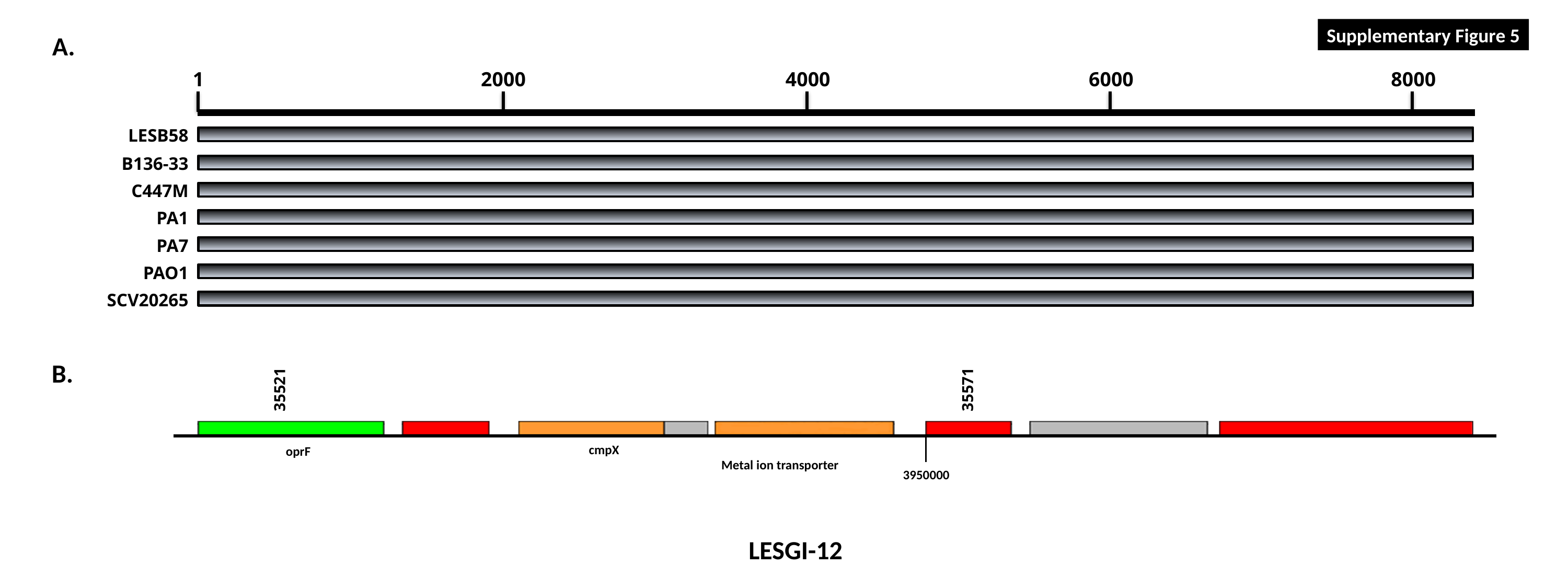

Supplementary Figure 5
A.
1
2000
4000
6000
8000
LESB58
B136-33
C447M
PA1
PA7
PAO1
SCV20265
35521
35571
cmpX
oprF
3950000
B.
LESGI-12
Metal ion transporter

## Slide 6
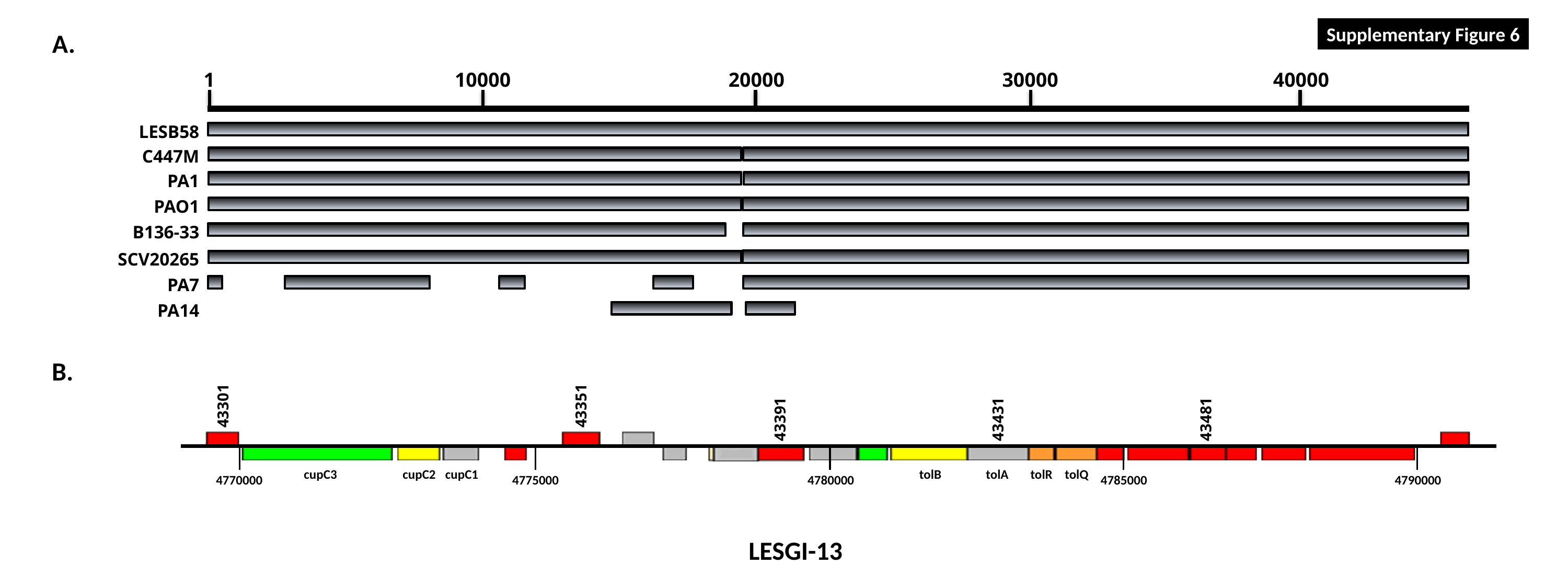

Supplementary Figure 6
A.
1
10000
20000
30000
40000
LESB58
C447M
PA1
PAO1
B136-33
PA7
PA14
SCV20265
43301
43351
43391
43431
43481
4770000
4775000
4780000
4785000
4790000
cupC3
cupC2
cupC1
tolB
tolA
tolR
tolQ
B.
LESGI-13

## Slide 7
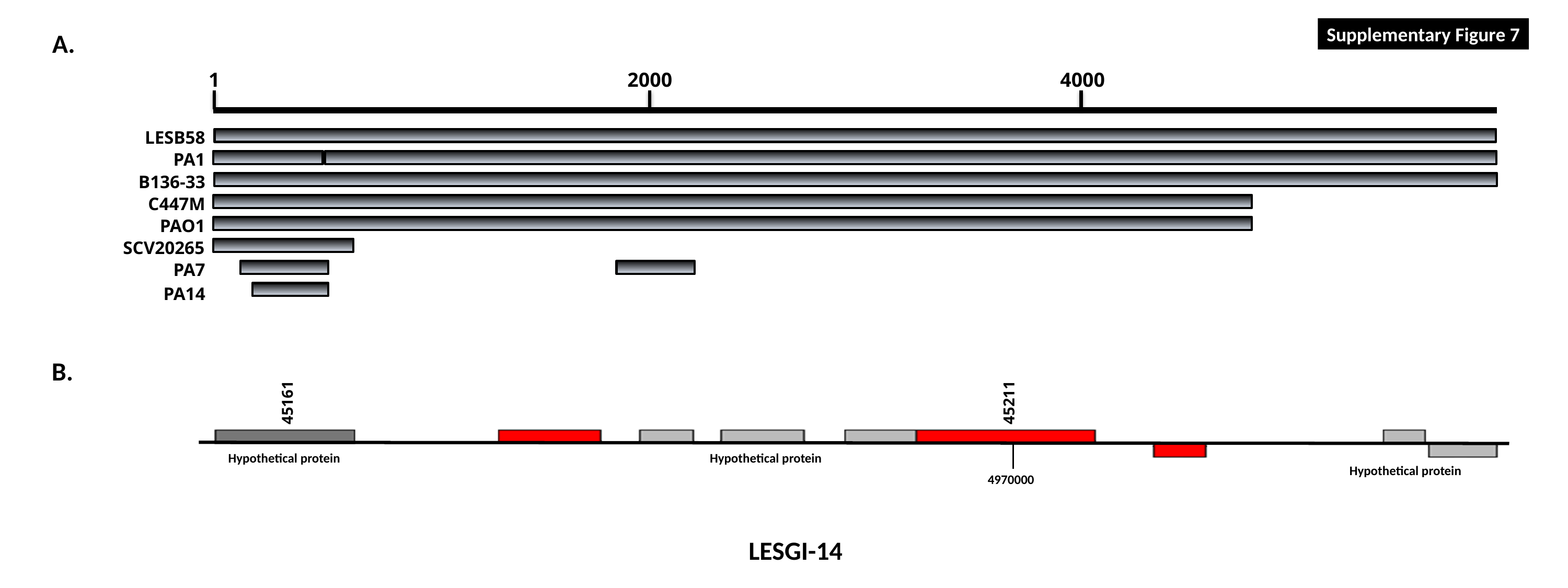

Supplementary Figure 7
A.
1
2000
4000
LESB58
PA1
B136-33
C447M
PAO1
SCV20265
PA7
PA14
45161
45211
4970000
B.
Hypothetical protein
Hypothetical protein
Hypothetical protein
LESGI-14

## Slide 8
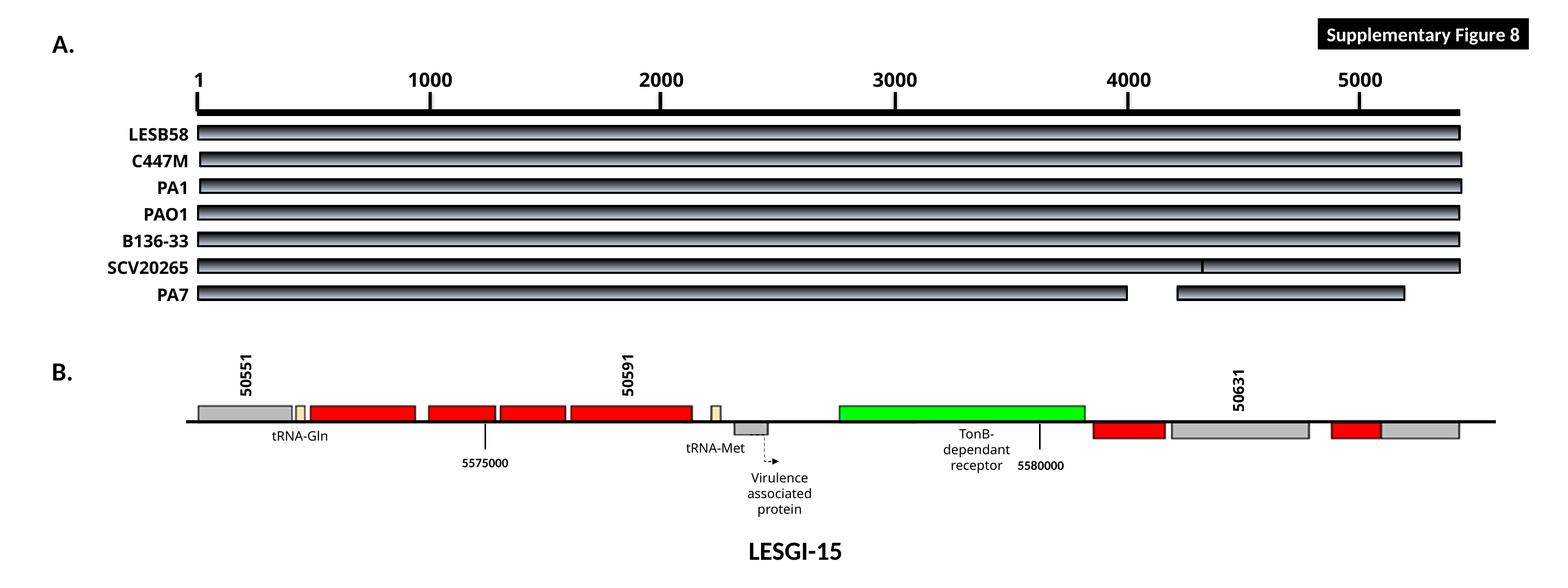

Supplementary Figure 8
A.
1
1000
2000
3000
4000
5000
LESB58
C447M
PA1
PAO1
B136-33
SCV20265
PA7
50551
50591
50631
5575000
5580000
TonB-dependant receptor
tRNA-Gln
tRNA-Met
Virulence associated protein
B.
LESGI-15

## Slide 9
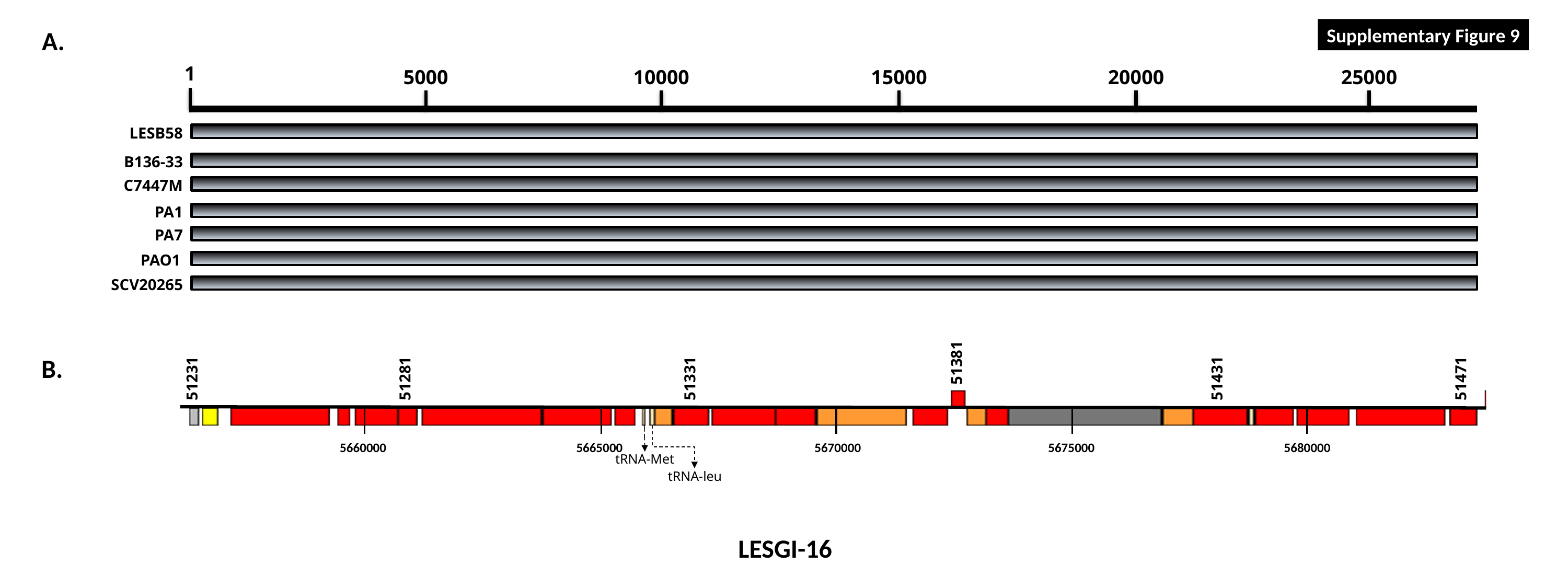

Supplementary Figure 9
A.
1
5000
10000
15000
20000
25000
LESB58
B136-33
C7447M
PA1
PA7
PAO1
SCV20265
51381
51231
51281
51331
51431
51471
5660000
5665000
5670000
5675000
5680000
tRNA-Met
tRNA-leu
B.
LESGI-16

## Slide 10
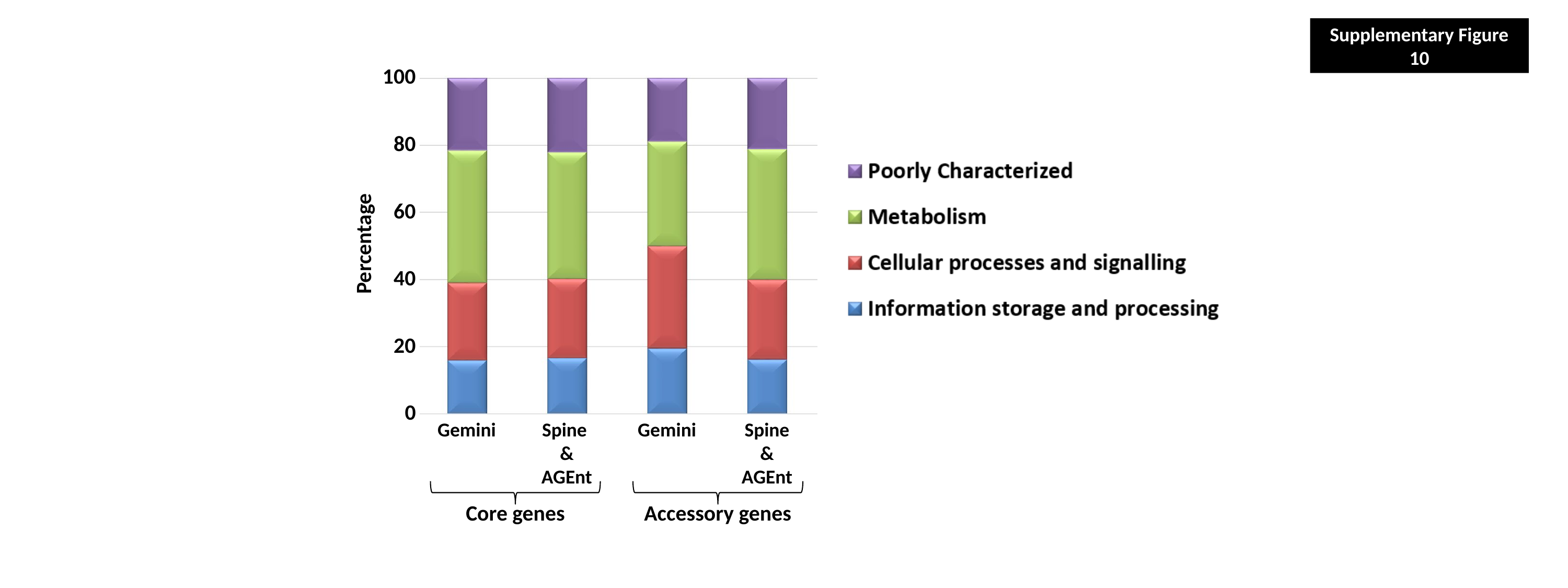

Supplementary Figure 10
100
80
60
40
20
0
Percentage
Gemini
Spine
&
AGEnt
Gemini
Spine
&
AGEnt
Core genes
Accessory genes
